# Characteristics of left atrial remodeling in patients with atrial fibrillation and hypertrophic cardiomyopathy in comparison to patients without hypertrophy

**DOI:** 10.1038/s41598-021-91892-y

**Published:** 2021-06-14

**Authors:** Sotirios Nedios, Borislav Dinov, Timm Seewöster, Frank Lindemann, Sergio Richter, Arash Arya, Nikolaos Dagres, Daniela Husser, Andreas Bollmann, Gerhard Hindricks, Andreas Müssigbrodt

**Affiliations:** 1 Department of Electrophysiology, Heart Center, University of Leipzig, Strümpellstr. 39, 04289 Leipzig, Germany; 2 Department of Cardiology, Cardiovascular Research Institute Maastricht (CARIM), Maastricht University Medical Center, Maastricht, The Netherlands; 3 Département de Cardiologie, Centre Hospitalier Universitaire de Martinique, BP 632, 97200 Fort de France, Martinique

**Keywords:** Cardiology, Interventional cardiology

## Abstract

Atrial fibrillation (AF) leads to remodeling characterized by changes in both size and shape of the left atrium (LA). Here we aimed to study the effect of hypertrophic cardiomyopathy (HCM) on the pattern of LA remodeling in AF-patients. HCM-patients (n = 23) undergoing AF ablation (2009–2012) were matched and compared with 125 Non-HCM patients from our prospective registry. Pre-procedural CT data were analyzed (EnSite Verismo, SJM, MN) to determine the maximal sagittal (anterior–posterior, AP), coronal (superior-inferior, SI and transversal, TV) dimensions and the sphericity index (LAS). Volume (LAV) was rendered after appendage (LAA) and pulmonary vein (PV) exclusion. A cutting plane, between PV ostia/LAA and parallel to the posterior wall, divided LAV into anterior- (LA­A) and posterior-LA (LA­P) parts. The ratio LA-A/LAV was defined as asymmetry index (ASI). HCM patients had a wider inter-ventricular septum and a smaller LV than Non-HCM patients. LA volume (LAV 166 ± 72 vs. 130 ± 36 ml, *p* = 0.03) and LA diameters were significantly larger in HCM patients. Anterior volume (LA-A: 112 ± 48 vs. 83 ± 26 ml, *p* < 0.001) differed significantly between groups, whereas the posterior volume LA-P (55 ± 28 vs. 47 ± 13 ml, *p* = 0.23) and LAS (75% vs. 78%, p = 0.089) was similar in both groups. As a result, ASI was significantly higher (67 ± 6 vs. 63 ± 6%, *p* = 0.01) in HCM than in Non-HCM patients. In conclusion, LA remodeling in patients with AF and HCM is characterized by asymmetric dilatation, driven by an anterior rather than a posterior dilatation. This can be characterized by three-dimensional imaging and could be used as surrogate of advanced atrial remodeling.

## Introduction

Atrial fibrillation (AF) is the most common arrhythmia with an increasing prevalence (currently 3%) in an aging population^1^. AF is associated with remodeling characterized by asymmetrical left atrial (LA) dilatation. This is particularly true for enlarged left atria, when due to anatomical constrictions there is a non-uniform LA dilatation. These changes represent an advanced AF stage and correlate with poor outcomes after catheter ablation (CA)^2–4^.

Hypertrophic cardiomyopathy (HCM) has prevalence of 0.2% and is the most common genetically determined cardiomyopathy. In contrast to the general population, almost one in four HCM-patients will develop AF during the clinical course of the disease. AF in HCM is associated with LA dilatation, clinical deterioration, increased mortality and adverse events^5–7^. However, hereto little is known about the anatomical LA changes associated with HCM. In the light of emerging disease-modifiers like Mavacamten (selective cardiac myosin ATPase inhibitor), such surrogates of disease progression could allow for close surveillance and response to therapy^8–10^.

To further investigate the relation between anatomical remodeling and hypertrophic cardiomyopathy, this study aimed to explore the differences in LA geometry in patients with HCM and those without left ventricular hypertrophy.

## Methods

### Patients

A total of 23 consecutive HCM patients with symptomatic AF, having undergone AF ablation procedures between 2009 and 2012, were matched with the use of a propensity score for clinical characteristics (based on the CHADS-VASc score) and compared for the LA anatomy with 125 patients without LV hypertrophy from the prospective Leipzig Heart Center Ablation registry. The diagnosis of HCM was based on two-dimensional echocardiographic evidence of a hypertrophied, non-dilated left ventricle (LV wall thickness ≥ 15 mm) and/or relevant outflow tract obstruction, in the absence of any other cardiac or systemic condition capable of producing such magnitude of hypertrophy^5^. In HCM patients with concomitant arterial hypertension, diagnosis of HCM was based on complementary criteria (i.e., LV outflow tract obstruction, magnitude or location of LVH) or the evidence of a mutation in gene analysis. Control patients had no LV hypertrophy. The institutional research committee of the University of Leipzig approved the study protocol and all data were collected in accordance with the Declaration of Helsinki and relevant guidelines.

### Echocardiography

All patients underwent a comprehensive transthoracic and transesophageal echocardiographic exam before catheter ablation according to the recommendations of the American and the European Society of Echocardiography^11^. Images were acquired at a designated echocardiography laboratory using a commercially available system (GE Healthcare Vivid 9, Philips Medical Systems iE33) equipped with a 3.5 MHz transducer. Intracardiac thrombi were ruled out. Recordings were made in parasternal long- and short-axis, as well as apical 4- and 2-chamber views with patients in the left lateral decubitus position. All images were ECG-triggered and stored for offline analysis. Left ventricular (LV) wall thickness, dimensions and LA diameter were measured in the left parasternal long axis (PLAX) view. The left ventricular ejection fraction (LVEF) was assessed by the modified Simpson’s method.

### Computed tomography

Cardiac-CT was performed before CA of the AF patients with a multi-detector 64-row helical system (Brilliance 64, Philips Medical Systems, Best, The Netherlands). Image acquisition was electrogram-gated and the parameters included: 70–120 kV, 850 mAs, 0.6 mm beam collimation, 0.625–1.25 mm thickness and 20–30 cm field-of-view. During an end-inspiratory breath-hold of 20 s, and following a timing bolus-chase injection (20 ml, 5 ml/s), 90 mL of an iodinated contrast medium (Ultravist 370, Bayer Vital, Germany) was administered. Finally end-systolic imaging data were recorded and used for three-dimensional (3D) reconstruction.

**Figure 1:**
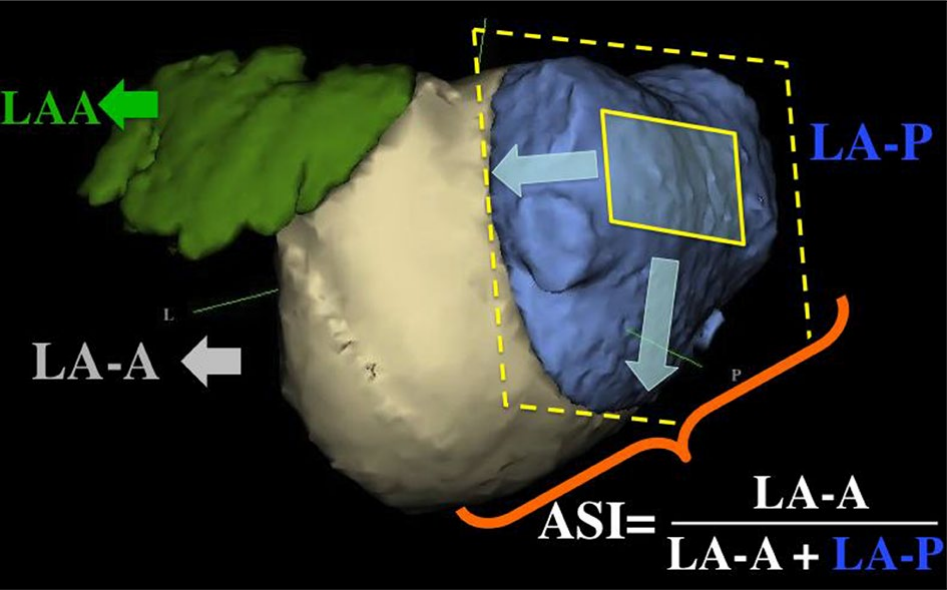
The asymmetry index (ASI) is the ratio LA-A/LAV. The division of LA volume (LAV) into anterior (LA-A) and posterior (LA-P) parts was defined by a cutting plane, between the pulmonary veins and the LA appendage (LAA) and parallel to the posterior wall^12^.

**Figure 2:**
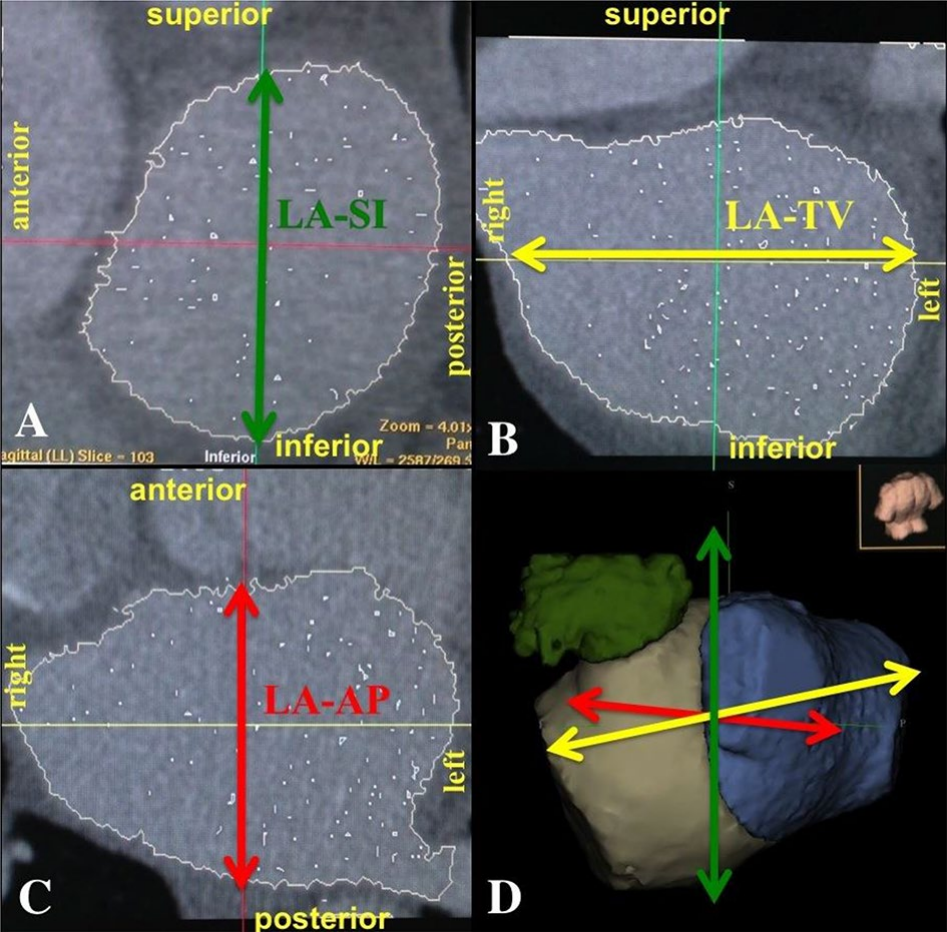
Left atrial diameters measured by Ensite Verismo: (**A**) supero-inferior and (**B**) transversal diameter on a coronal plane, (**C**) antero-posterior on a sagittal plane and (**D**) 3-D LA model.

CT data were reviewed using 3D volume rendering (EnSite Verismo, SJM, MN). Left atrial volume (LAV) after exclusion of the atrial appendage (LAA) and the pulmonary veins (PV) was determined by LA area summation. The LAV was then divided by a cutting plane, between the anterior segment of the PV ostia and the atrial appendage and parallel to the posterior wall. The resulting anterior (LA-A) and posterior (LA-P) partial volumes were calculated and the ratio LA-A/LAVx100 was defined as the asymmetry index (ASI, % Fig. 1)^12^. The LA diameters were also measured (Fig. 2). The standard variation and the average radius (AR) of the LA was then calculated from these diameters, in order to calculate the left atrial sphericity as previously described; LAS = 1 − CVS*100, with CVS representing the coefficient of variation defined as CVS = AR standard deviation/AR^3,13^. Image analysis was performed offline by an experienced observer blinded to ablation data and the patient's follow-up. To avoid un-blinding of HCM patients by the septum thickness, LA was segmented and analyzed separately. Initial measurements of 20 random patients were repeated 4 weeks later by the same investigator and a second reviewer in a blinded fashion.

### Ablation procedure and follow-up

All patients gave an informed consent for the catheter ablation procedure. Under continuous sedation and hemodynamic monitoring, left atrial access was obtained through a transseptal puncture. Heparin was administered intravenously to achieve a target ACT over 300 s. A circumferential pulmonary vein isolation using irrigated catheters was successfully performed in all patients. Electroanatomical mapping systems (Carto 3; Biosense Webster, Diamond bar, CA or EnSite Velocity; Endocardial Solutions, St. Paul, MN) were used for creation of the pulmonary veins and LA anatomy and for visualization of the catheters. After completion of the circumferential ablation, voltage maps of the LA in sinus rhythm were constructed and the areas showing low-amplitude signals (< 0.5 mV) were annotated as low-voltage areas. Additional linear ablation was performed in these areas to connect them with electrically unexcitable hallmarks as previously described^12^. Follow-up was performed with regular clinical examinations and 7-day-Holter ECG recordings (Lifecard CF, Delmar-Reynolds Medical Inc, Irvine, CA, USA) at 6, 12, 24 and 36 months of follow-up. AF recurrence was defined as an AF episode or a LA macro-reentry tachycardia of ≥ 30 s documented in ECG. Episodes occurring during an initial 3-month blanking period after the ablation were not included in the analysis. In case of recurrence, AADs were adapted and a repeat ablation was considered on an individual basis.

### Statistics

Continuous variables are expressed as mean and standard deviation (SD). Categorical variables are reported as frequencies and percentage. Kolmogorov-Smirnoff test was used to analyze the distribution of continuous variables. On that basis, parametric variables were compared by means of paired Student’s t-test (for 2 groups) and non-parametric variables by Wilcoxon-test or chi-square test. Intra-observer and inter-observer variability was assessed with Pearson’s r-values^2^. Clinical variables and LA measurements were evaluated with univariate cox regression analysis to determine their association with AF recurrence. Variables with *p* < 0.1 were included in a forward multivariate model to determine the hazard ratio (HR) and the confidence interval (CI) of independent AF recurrence predictors. Recurrence rate was depicted with Kaplan–Meier curves. A two-tailed *P* ≤ 0.05 was considered statistically significant. Analysis was performed with SPSS 21.0 (SPSS Inc., Chicago, USA).

## Results

### Baseline characteristics

The patients’ characteristics are displayed in Table 1. HCM patients had a bigger inter-ventricular septum and a smaller LV than Non-HCM patients, but all other clinical characteristics were similar between groups. The frequency of pacemakers was similar between groups, but ICDs were more common among HCM patients (8% vs. 4%).

**Table 1:** Characteristics of patients with and without hypertrophic cardiomyopathy (HCM).

Baseline characteristics	HCM	Non-HCM	*P*
Number of patients, n	23	125	
Age, years	57 ± 8	59 ± 9	0.15
Males, n (%)	16 (70)	94 (75)	0.37
Body mass index, kg/m2	29 ± 5	29 ± 6	0.83
Persistent AF, n (%)	13 (57)	62 (50)	0.35
CHADS-VASc Score, n	1.4 ± 0.9	1.8 ± 1.3	0.14
Hypertension, n (%)	17 (74)	89 (71)	0.51
Diabetes, n (%)	3 (13)	17 (14)	0.62
Coronary artery disease, n (%)	3 (13)	16 (13)	0.59
Stroke history, n (%)	0 (0)	6 (5)	0.59
Pacemaker, n (%)	2 (9)	5 (4)	0.93
Defibrillator (ICD), n (%)	8 (35)	5 (4)	0.001
Morrow resection or TASH, n (%)	8 (35)	–	0.001
**Echocardiography**			
LV-EF, (%)	57 ± 10	60 ± 7	0.18
LA diameter, mm	46 ± 8	43 ± 6	0.05
LV end-diastolic diameter (EDD), mm	45 ± 5	50 ± 7	0.003
Interventricular septum diameter, mm	19 ± 4	12 ± 2	< 0.001
**Computed tomography**			
LA-AP (antero-posterior), mm	53 ± 10	47 ± 9	0.006
LA-SI (supero-inferior), mm	67 ± 10	62 ± 7	0.03
LA-TV (transversal), mm	82 ± 14	76 ± 8	0.08
LAA volume, ml	11 ± 5	8 ± 3	0.02
LA volume, ml	166 ± 72	130 ± 36	0.03
LA-A volume anterior, ml	112 ± 48	83 ± 26	< 0.001
LA-P volume posterior, ml	55 ± 28	47 ± 13	0.23
Asymmetry index (LA-A/LAV), %	67 ± 6	63 ± 6	0.01
Average Radius, mm	67 ± 10	62 ± 6	0.01
LA sphericity (LAS), %	78.1	75.3	0.089
Number of ablations, n	1.4 ± 0.7	1.3 ± 0.6	0.27
Additional substrate modification, n (%)	14 (61%)	63 (50%)	0.37

Within the HCM cohort, there were eight patients (35%) with LV outflow tract obstruction. Seven of these patients had previously undergone a Morrow resection or a trans-coronary ablation of septal hypertrophy (TASH). Genetic analysis was performed in five patients (22%) and was positive in three patients; two with different heterozygote mutations in MYH7 and one with a heterozygote mutation in MYBPC3. The apical form of HCM was diagnosed in two patients (9%).

### LA geometry and hypertrophic cardiomyopathy

The intra- und inter-observer correlation of LA measurements (LAV, LA-A, LA-P and ASI) was found to have coefficients of 90 ± 2%, as previously published^12^. LA volume (LAV 166 ± 72 vs. 130 ± 36 ml, *p* = 0.03) and diameters (LA-AP: 53 ± 10 vs. 47 ± 9 mm, *p* = 0.006; LA-SI: 67 ± 10 vs. 62 ± 7, p = 0.03) were significantly larger in HCM patients, but the difference in sphericity did not reach statistical significance (LAS 75.3% vs. 78.1%, *p* = 0.089). Anterior volume (LA-A: 112 ± 48 vs. 83 ± 26 ml, *p* < 0.001) differed significantly between groups, whereas the posterior volume LA-P (55 ± 28 vs. 47 ± 13 ml, *p* = 0.23) was similar in both groups. As a result, ASI was significantly higher (67 ± 6 vs. 63 ± 6%, p = 0.01) in HCM than in Non-HCM patients. In order to evaluate the effect of LA dilatation on ASI, we performed an additional (1:2) matching process for LA volume and found that the resulting groups had different ASI (67 ± 6 vs. 64 ± 5%, *p* = 0.03) despite similar LAV (166 ± 72 vs. 152 ± 50 ml, *p* = 0.45) and LAS (77.6% vs. 78.1, *p* = 0.73).

### Predictors of recurrence

**Figure 3:**
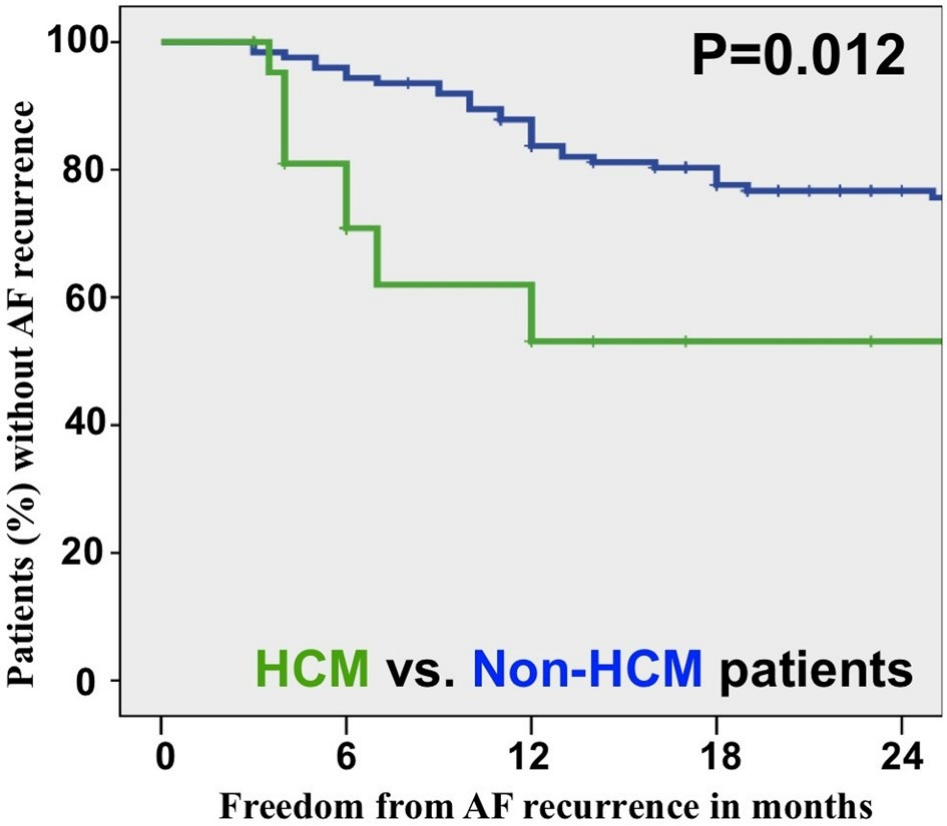
Kaplan–Meier curve depicts the time to first recurrence after last ablation. AF, atrial fibrillation; HCM, hypertrophic cardiomyopathy.

In the Non-HCM group, cox regression analysis revealed that LA volume and ASI were predictors of AF recurrence, whereas sphericity was not (*p* = 0.71). After multivariate adjustment, ASI was the only independent predictor of SR stability (HR = 1.08, CI = 1.008 to 1.157, *p* = 0.03). In Non-HCM patients, LAV had a weak correlation with ASI (r = 0.24, *p* = 0.013) and LAS (r = 0.24, *p* = 0.012), but there was no significant correlation between ASI and LAS (*p* = 41). The correlation between ASI and LAV was even weaker in the total cohort (r = 0.2, *p* = 0.022) and there was no significant multi-collinearity detected in the cox-regression model when using variance inflation factor for the probability of survival (VIF = 1.06). In the HCM group, no parameter reached statistical significance due to the limited number of patients. The number of ablations and the frequency of additional lesions were similar between the two groups (Table 1). The Kaplan–Meier curves are depicted in Fig. 3.

## Discussion

### Main findings

This study aimed to evaluate the effect of hypertrophic cardiomyopathy (HCM) on the pattern of LA remodeling in AF-patients. We found that in patients with HCM the anatomical remodeling of the left atrium is described by asymmetric dilatation (more pronounced anteriorly), a pattern that has been previously associated with advanced AF and poor outcomes after catheter ablation^2–4^. Therefore, changes in atrial geometry could help us understand to what extent the HCM has strained the left atrium leading to a remodeling associated with persisting or recurrent AF. This could be used to guide or evaluate the response to emerging modifying therapies^8–10^.

### Atrial remodeling and hypertrophic cardiomyopathy

Our findings supplement previous studies and emphasize the association between HCM and LA remodeling^14^. LA size and fibrosis are known predictors of recurrences after CA^14,15^. PV reconnection has been shown to be very frequent in the context of HCM resulting in modest long-term success^16^. Additionally, PV re-isolation in HCM may not improve prognosis as in Non-HCM patients^17^. Later AF recurrences in HCM patients have been associated with non-PV triggers may cause^18^. Recent advances in technology though have resulted in more durable lesions with better procedural and follow-up results^19^. Together with emerging modifying drug therapies like Mavacamten^8–10^, these findings argue for early intervention before advanced atrial remodeling takes place^20,21^.

The asymmetric LA dilatation found in our study underscores the interaction between left ventricle and left atrium, especially during diastole, when LA is directly exposed to LV pressures. HCM is commonly accompanied by diastolic dysfunction and myocardial fibrosis that results in increased LA pressures and progressive chamber enlargement^22,23^. This is further supported by our recent findings that emphasize the pathophysiological importance of diastolic dysfunction on the asymmetric LA remodeling in patients without HCM^12^.

Additionally, the typical tissue architecture in HCM with fibrosis, disarray of myocardial cells and pronounced electrical anisotropy could make the substrate more vulnerable to geometrical changes^24,25^. Since HCM patients have more prominent anterior dilation that potentially increases the surface of the arrhythmogenic substrate, additional ablation, as e.g. alcohol ablation of the vein of Marshall vein, may be an approach that could be considered in these patients. This hypothesis though remains to be evaluated by further clinical studies.

**Figure 4:**
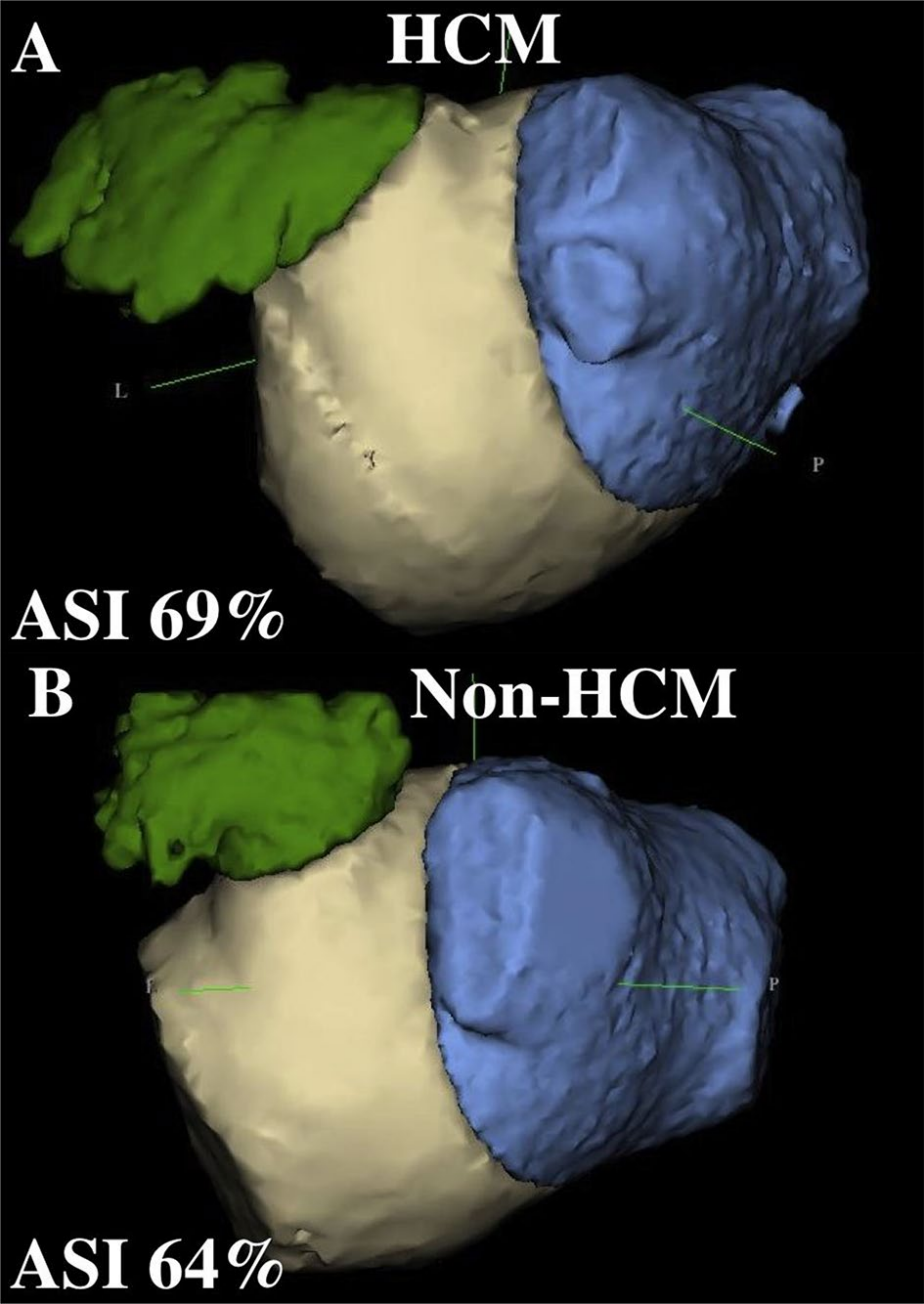
Asymmetry index (ASI) in a patient with (**A**: ASI 69%, LAV 130 ml) and one without (**B**: ASI 64%, LAV 125 ml) hypertrophic cardiomyopathy.

In the present HCM cohort, atrial dilatation has shown an asymmetric pattern that may not be seen in the standard echocardiographic evaluation of the patient. This pattern was present even after adjusting for LAV dilatation. The anatomical discrepancies between HCM and Non-HCM groups were far more pronounced than one would expect according to the LA diameters. This suggests that the LA diameter, measured in parasternal long axis view, which is currently often used to characterize LA anatomy prior to AF treatment, is an insensitive parameter for LA anatomical remodeling and imperfectly characterizes the complex changes occurring in the atria of HCM patients with AF (Fig. 4). Similarly insensitive was the sphericity index, which was previously suggested as a surrogate of LA remodeling^3^. In the current cohort LAS was not different between HCM and Non-HCM patients and did not predict AF recurrence. This is in congruence with recent findings that revealed LA sphericity to be unsuitable as a marker of AF-related atrial remodeling^13^.

### Clinical implications

AF in HCM is associated with a substantial risk for heart failure–related mortality, stroke, and severe functional disability^5–7^. Patients with HCM and AF often need more procedures and frequently additional antiarrhythmic drug therapy in order to maintain sinus rhythm. Therefore, it is crucial to improve rhythm outcomes in those patients, by e.g. a better therapy selection and an individual ablation strategy.

The present study illustrates the characteristics changes of LA remodeling in HCM patients with AF, when compared with Non-HCM AF patients. We found a specific asymmetric pattern that could be overseen with echocardiography. Since LA anatomy is an important predictor of rhythm stability after catheter ablation^26^ and could even improve risk prediction or thromboembolic events^27^, our findings urge for a more comprehensive 3D evaluation of the anatomical LA changes in HCM patients, in order to improve patient selection and the appropriate ablation approach. In patients with normal atria for example, cryoablation could be an acceptable first option, whereas in patients with pronounced LA changes, radiofrequency ablation, pulsed field ablation and possibly alcohol ablation of the vein of Marshall would be better to target an advanced AF substrate. Additionally, since Mavacamten has been shown to reduce hypertrophy and LA volumes in patients with obstructive HCM, it could be used to support reverse remodeling and allow for ablation of patients that may have otherwise been excluded. Thus surrogates of disease progression like LA volume and symmetry (ASI) could allow for close surveillance and response to therapy^8–10^. These results are hypothesis-generating and call for more studies testing these parameters by new ablation or medical strategies to clarify the optimal treatment in HCM patients.

### Limitations

This is a post-hoc analysis of a selected patient population with symptomatic AF and HCM compared to patients without LV hypertrophy. The sample size of the HCM group was limited and could not be extended for the purpose of this study due to institutional change of the pre-procedural imaging modality from CT to MRI. This reduced the statistical power and precluded a regression analysis in the HCM group. Certainly, higher ASI could be partially attributed to larger LAV. Although ASI emerged as an independent recurrence factor after adjustment for LAV in the Non-HCM group, we were not able to test this in the HCM group. Nevertheless, Kaplan–Meier curves could demonstrate the significantly worse rhythm outcomes of HCM patients when compared to Non-HCM patients. Thus the study remains informative despite the small cohort size.

Since most of the patients had persistent AF, diastolic dysfunction could not be sufficiently quantified and its association with the specific asymmetric LA dilatation could not be further elucidated. Calculation of ASI and LAS by computed tomography required radiation exposure and contrast injection, which prohibited follow-up studies that could reveal the effect of ablation on LA asymmetry. However, ASI is an easily obtained pre-procedural parameter and since newer techniques, such as magnetic-resonance tomography or 3D echocardiography are entering the clinical routine, they could potentially be obtained without radiation in the future. In summary, our findings may be viewed as generating new hypothesis that could be further examined in future studies.

## Conclusion

LA remodeling in patients with AF and HCM is characterized by asymmetric dilatation, driven by an anterior rather than a posterior dilatation. This can be characterized using by 3D three-dimensional imaging and could be used as surrogate of advanced atrial remodeling.
